# Multipotent Mesenchymal Stromal Cells: Possible Culprits in Solid Tumors?

**DOI:** 10.1155/2015/914632

**Published:** 2015-07-27

**Authors:** Pascal David Johann, Ingo Müller

**Affiliations:** ^1^Division of Pediatric Neurooncology, German Cancer Research Center, Im Neuenheimer Feld 580, 69120 Heidelberg, Germany; ^2^Division of Pediatric Oncology, Hematology, Immunology and Pneumology, University Children's Hospital, Im Neuenheimer Feld 430, 69120 Heidelberg, Germany; ^3^Department of Pediatric Hematology and Oncology, University Medical Centre Hamburg Eppendorf, Martinistrasse 52, 20246 Hamburg, Germany

## Abstract

The clinical use of bone marrow derived multipotent mesenchymal stromal cells (BM-MSCs) in different settings ranging from tissue engineering to immunotherapies has prompted investigations on the properties of these cells in a variety of other tissues. Particularly the role of MSCs in solid tumors has been the subject of many experimental approaches. While a clear phenotypical distinction of tumor associated fibroblasts (TAFs) and MSCs within the tumor microenvironment is still missing, the homing of bone marrow MSCs in tumor sites has been extensively studied. Both, tumor-promoting and tumor-inhibiting effects of BM-MSCs have been described in this context. This ambiguity requires a reappraisal of the different studies and experimental methods employed. Here, we review the current literature on tumor-promoting and tumor-inhibiting effects of BM-MSCs with a particular emphasis on their interplay with components of the immune system and also highlight a potential role of MSCs as cell of origin for certain mesenchymal tumors.

## 1. Introduction

Although multipotent mesenchymal stromal cells were first described in the context of regenerative medicine in the early 1970s, further research could reveal remarkable features other than their plasticity towards the osteogenic, chondrogenic, and adipogenic line [1, 2]. Particularly their immunosuppressive potential has gained widespread attention and paved the way to their application in a variety of immune disorders such as Graft-versus-Host Disease or multiple sclerosis [3, 4]. A growing body of literature in the last years has focused on a potential role of MSCs in malignancies, covering mainly two aspects: MSCs as a potential cell of origin for certain mesenchymal tumors on the one hand and the interplay of MSCs with different components of the tumor microenvironment on the other hand. These issues are of pivotal importance as many experimental oncological therapies employ MSCs as cellular vehicles that migrate to tumor sites. In order to fully grasp the interplay of MSCs with the tumor microenvironment, it is necessary to shed light on the different cells which constitute the stroma of solid tumors.

## 2. The Tumor Microenvironment: A Complex Niche

In 1986, Dvorak highlighted the similarities between neoplastic and inflammatory tissue, thus founding the perception of tumors as “wounds that do not heal” [5]. This comparison is based on many similarities between inflammation and carcinogenesis, which include the recruitment of a variety of immune effector cells and mesenchymal cells such as tumor associated fibroblasts [6] (see Table 1 for an overview on different components of the tumor microenvironment).

Literature of the last years has added important functional aspects to the (in earlier times primarily histological) description of the tumor stroma. Among the first immune cells for which functional polarizations have been reported are macrophages: The M1 and M2 subclassification refers to macrophages that have acquired different properties depending on their previous exposure to cytokines: Roughly, the M1 macrophage has been associated with a response to stimuli from Th1 cells, while the M2 subtype is being induced by IL-4 and has been ascribed to inhibit immune cell proliferation rather than eliciting an antitumor response.

Additionally, macrophages participate in restructuring the tumor extracellular matrix by the secretion of matrix metalloproteinases and growth factors (reviewed in [7]). Thus they also interact with tumor associated fibroblasts, which secrete TGF-*β*, SDF-1, and other growth factors both in wounds and in tumors.

While the induction of this tumor-suppressive macrophage subtype represents the most commonly accepted functional and phenotypic change of an immune cell that enters a solid tumor, other effector cells have also been demonstrated to undergo functional changes upon interaction with the tumor microenvironment: Reduction in the expression levels of activating receptors such as NKp30 and NKp46 is a consequence of NK cell and tumor cell interaction in several entities [8, 9]. The ratio of CD56^bright^ CD16^low^ to CD56^dim⁡^ CD16^+^ cells has been found to be shifted towards the less mature, first subtype in NSCLC [8]. Beyond the impairment of NK cell effector function, the influence of the tumor microenvironment may even reprogram NK cells towards a proangiogenic phenotype [9]: NK cells could be demonstrated to secrete angiogenic factors such as VEGF or PDGF-*α* which was associated with a worse prognosis in certain malignancies [10].

Other immune cells such as dendritic cells have also been reported to be compromised by the tolerogenic tumor microenvironment: Being exposed to factors such as being secreted by the tumor microenvironment, dendritic cell differentiation can be arrested in an immature state and are then enabled to induce regulatory T cells by the secretion of IL-10 and TGF-*β*, thus further impairing the antitumor response.

In summary, for most immune cells a polarization into a tumor-suppressive and a tumor-promoting/effector function impaired phenotype has been documented and there is substantial evidence that the tumor microenvironment compromises effector functions at various levels.

Before specifically considering the interaction of fibroblasts/tumor associated MSCs with the tumor microenvironment, a further classification of these stromal cells is necessary as many publications do not present a clear phenotypic characterization but rather focus on functional properties of fibroblastoid cells, derived from tumors.

## 3. From Mesenchymal Stromal Cells to Tumor Associated Fibroblasts: Two Sides of One Coin?

Multipotent mesenchymal stromal cells can be isolated from a variety of tissues such as bone marrow, adipose tissue, Wharton's jelly, peripheral blood, and others [11]. Despite this plethora of origins, the phenotypic similarities between MSCs enabled the formulation of consensus definition criteria for MSCs. They consist of a set of phenotypic markers (such as CD73, CD90, and CD105) and include the capability to differentiate into osteoblasts, chondrocytes, and adipocytes [12]. While tissue MSCs from different backgrounds meet these criteria [13], it is hitherto unclear to what extent all these features are shared by other mesenchymal cells from the same tissue: Dermal fibroblasts for instance have also been shown to exhibit a trilineage differentiation potential [14]. The same seems to be true for other mesenchymal cells which share surface markers such as CD90 and CD105 with MSCs (Table 2), [15]. Despite this overlap in phenotypic properties, more recent studies could identify markers that are able to separate MSCs from fibroblasts such as CD106 which displays specific expression on MSCs and is absent on their fibroblast counterparts [16].

With regard to the tumor microenvironment, a comprehensive characterization of TAFs from different cancer entities aiming at the establishment of marker to discern each cell type is still missing. Spaeth et al. could demonstrate that the coculture of MSCs with supernatant from tumor cells could induce activation markers (FAP, TSP1, and *α*-SMA) that are typical for TAFs [17]. However, this quantitative difference in expression of TAF-associated proteins is not sufficient to qualify the aforementioned markers as a specific marker for either TAFs or MSCs. Paunescu et al. have systematically compared the expression of MSC marker molecules (such as CD44, CD90, and CD73) between MSCs, TAFs, skin fibroblasts, and HDFa and found no differences in the expression levels of these molecules [18]. Moreover, other less established MSC markers such as vimentin were also found to be commonly expressed in all fibroblastoid cell types [19].

Consistent with the results of Osonoi et al., in this and other studies, TAFs were also shown to display plasticity towards the osteogenic line.

The sole functional property distinguishing TAFs and MSCs was a higher proliferative capacity and cytokine production of TAFs when compared to BM-MSCs [19].

In absence of a single surface marker to discern TAFs and MSCs, gene expression profiling may be the only way to pinpoint the differences between MSCs and fibroblasts from cancer [20].

Given this dearth of specific markers, further evidence is needed to clarify whether both cell types are essentially identical or coexisting cell types within the tumor stroma. In the following passage we focus on studies which specifically examine bone marrow derived MSCs or MSCs isolated from tumors.

## 4. MSCs and Their Interplay with Components of the Tumor Microenvironment

Several aspects of MSC biology have been examined in recent literature: One approach aims at assessing the effect of tumors BM-MSCs after coinjection with subcutaneous or orthotopic xenograft tumors. The careful examination of changes in tumor growth pattern and in its microenvironmental structure after exposure to MSCs is pivotal given the numerous therapeutic studies that use MSCs for tissue regenerative and other purposes.

Another class of studies has characterized MSCs or tumor associated fibroblasts that were isolated directly from primary tumor tissue and is mainly dedicated to studying the functional effects of tumor derived MSCs on immune cells.

## 5. The Immunomodulatory Role of MSCs and TAFs in the Context of Solid Tumors

A functional feature which has fuelled immunological and oncological research is the immunosuppressive property of BM-MSCs. They are capable of inhibiting the proliferation of T cells in PBMC (peripheral blood mononuclear cells) preparations* in vitro* and* in vivo*.

At a more detailed level, this property expands to almost all effector cells of the peripheral blood such as T cells, B cells, and NK cells that are inhibited not only in their proliferative capacity, but also in cytolysis and antibody production.

This immunosuppression at a cellular level is widely considered to be the functional basis for the systemic effects, for example, in Graft-versus-Host Disease. This property involves several soluble factors such as galectin-3, galectin-9, indoleamine 2,3-dioxgynease (IDO), IL-10, and HLA-G [21].

With regard to the neoplastic context, the infiltration of immune cells to solid tumors is a well-described phenomenon [8].

There is growing evidence that T-MSCs share these antiproliferative and immunosuppressive functions with their bone marrow counterparts: In an* in vitro* study in human gliomas, Ochs et al. could show that MSC-like pericytes display inhibitory functions on CD4^+^ T cells similar to BM-MSCs [22]. This effect was found to be mediated by prostaglandin-E2 and HGF which have also been implicated in the immunosuppression exerted by BM-MSCs.

More recently, the glioma promoting effect of pericytes has been validated in a xenograft model of this disease, supporting the notion that these mesenchymal cells can switch from a tumor-suppressive phenotype to a tumor-promoting one [23].

Notably, the antiproliferative effect of MSCs also affects microglia cells which represent the quantitatively most important immune cell population of the brain. Proliferation of these cells is impeded by a mechanism that involves tumor necrosis factor *α* (TNF-*α*) [24].

Not only MSCs isolated from brain tumors but also MSCs derived from pediatric malignancies and from colorectal carcinomas display immunosuppressive properties and are able to downregulate activating NK cell receptors and impair the tumor lysis by NK cells* in vitro* [22, 25].

Montesinos et al. have drawn a direct comparison between MSCs from nonneoplastic cervical tissue and cervical cancer demonstrating an identical marker profile of both MSC types yet with distinct functional properties: Production of the immunosuppressive interleukin IL-10 was markedly increased in tumor associated MSCs, underpinning a role in establishing an immunosilenced, quiescent niche [26].

To summarize, the majority of publications state a tumor-promoting effect by suppression of immune effector cells. Hereby, the factors which have been identified as mediators of these effects are by and large the same as the ones implicated in BM-MSC immunosuppression (i.e., IDO, PGE2, and others).

Few publications contrast with these observations; Barnas et al. found that TAFs could induce CD3/CD28 depending activation of T cells and could not confirm an inhibition of these effector cells [27].

Taking the results of all these functional studies together, inhibitory effects on various immune effector cells have been demonstrated by a number of studies (Table 3).

The abundance of these publications however needs to be taken with a grain of salt: Very few experimental designs aim at a side-by-side comparison of tumor derived MSCs with fibroblasts or MSCs from adjacent healthy tissue. Given the fact that also fibroblasts from different other tissues seem to share the antiproliferative property on immune cells [15], it is questionable whether the sole measurement of effector cell proliferation or receptor expression status without an adequate control population is able to faithfully recapitulate the situation in the tumor. While the mechanism of immunosuppression is well documented, both for T-MSCs and for BM-MSCs, a comprehensive comparison with other fibroblastoid cells from nonneoplastic tissue is still missing.

## 6. The Homing of BM-MSCs in Solid Tumors

Aside from tissue MSCs that are already present at sites of tumorigenesis and that may undergo a differentiation to TAFs, there is substantial evidence that MSCs from the bone marrow may also home in the tumor thus contributing to the tumor stroma: This ability of MSCs has been addressed by a number of studies aiming at the therapeutic use of this property ([28], also reviewed in [29]).

A seminal study by Quante et al. could show that in the setting of inflammatory gastric cancer about 20% of TAFs originate from bone marrow MSCs [30]. The chemoattraction of BM-MSCs to the tumor in these studies was mainly governed by TGF-*β* and SDF-*α*, factors which have previously been shown to be secreted by various components of the tumor microenvironment [6]. By gene expression profiling, higher levels of inflammation-associated genes were found to be expressed in these bone marrow derived TAFs than by their bone marrow counterparts.

The exact proportion of mesenchymal cells in tumors that originate from the bone marrow may vary: In a study of ovarian cancer as much as 60–70% of the stroma could be traced back to BM-MSCs [31].

Notably, the majority of publications (for an overview please see Table 4) that could demonstrate a migration of MSCs to the tumor are conducted in adult cancers and carcinomas with a generally high proportion of mesenchymal cells (e.g., pancreatic cancer). The migration pattern of BM-MSCs to, for instance, pediatric, neoplasias remains largely understudied.

When trying to dissect the mechanism by which BM-MSCs are attracted to the tumor, most of the studies identify inflammatory cytokines as important mediators (such as SDF-1 or CXCR6). Hence the same molecules which have been identified as mediators of immune cell attraction to neoplasms are also identified as major protagonists in the context of BM-MSC homing. However, this may also be an effect of focusing the analysis to a set of well-documented factors (more comprehensive proteomic analyses from tumor supernatants are needed to identify other factors that may mediate the migration).

Teo et al. have shed more detailed light on the migratory mechanism by which MSCs overcome the endothelial barrier in inflammatory and cancer microenvironments: Similar to leukocytes, these cells are able to perform para- and transcellular diapedesis from the blood vessel lumen to the tumor [32].

Even in brainstem glioma models, intravenous administration of TRAIL-expressing MSCs resulted in increased apoptosis in the tumors which correlated with a significantly increased survival [33], indicating that even the blood brain barrier can be overcome. The systematic exposure of adipose tissue derived MSCs to laminin, fibronectin, and glioma-conditioned media was able to increase to rate of MSCs homing in a rodent model of glioblastoma [34].

## 7. Paracrine Effects of BM-MSCs and Their Role in Extracellular Matrix Remodeling within Cancers

The establishment of different homing mechanisms of MSCs in tumors prompts the question about the role of these cells after having infiltrated the tumor.

In the case of breast cancer, otherwise weakly metastatic tumor cells greatly increased their metastatic potential when stimulated by MSCs. Mechanistically, the secretion of CCL5 by MSCs seemed to be a crucial factor in this process. Notably, the effect relied on the constant production of this chemokine and was reversible when BM-MSCs and tumor cells were separated after short, initial priming.

In keeping with this finding in breast cancer, Xu et al. could demonstrate that the frequency of metastases in a human osteosarcoma model is increased, when they injected MSCs intravenously after xenografting the tumor [35, 36]. Here again, CCL5 was at least partly responsible for this effect. Another mechanism by which MSCs may increase tumorigenesis of breast cancer cells is the induction of lysyl oxidase which was shown to enhance the metastatic potential of breast cancer cells in xenografts [37].

Particularly when it comes to bone marrow metastases of breast cancer, MSCs could exert detrimental effects as they are able to promote the transmigration of breast cancer cells over endothelia* in vitro* [38].

Another link between tumor progression and MSCs was established in a model of hepatocellular carcinoma (HCC), in which the tumor growth promoting effect was strongly dependent on the presence of TGF-*β* secreted by MSCs [39]. The important role of the cytokine TGF-*β* in the tumorigenic effect of MSCs is further highlighted by experiments from Shangguan et al. work: By transducing MSCs with activin membrane-bound inhibitor, a TGF-*β* receptor with inactive cytoplasmatic domain, a repression of the TGF-*β* axis could be achieved and the tumor protective properties of MSCs in a breast cancer model could be abrogated [40].

It is remarkable that these studies seem to converge on a relatively small set of molecules that have previously been studied in either the inflammatory context or the context of chemoattraction/immunosilencing in the tumor microenvironment. Here again, it is not clear whether the set of factors which has been examined is limited and other substances may also play a role, or whether the inflammation-related chemokines (such as CCL5) just play ubiquitous role in chemoattraction of multiple cells.

Apart from providing growth stimuli by paracrine effects, the remodeling of the extracellular matrix is another aspect by which mesenchymal cells are able to facilitate tumor progression: MMP 13 (matrix metalloproteinase 13) has been shown to promote cancer cell invasion* in vitro* and is overexpressed in mesenchymal stem cell like myofibroblasts in solid tumors [41].

A series of studies has attempted to mimic the low oxygen tension in solid tumors, thus by exposing BM-MSCs to low oxygen tensions: Potier et al. could demonstrate that temporary hypoxia of MSCs lead to a twofold increase in the secretion of vascular endothelial growth factor (VEGF), whereas the plasticity of MSCs towards the adipogenic and osteogenic line is reduced under hypoxia as it was shown by Holzwarth and coworkers [42, 43]. A potential role of VEGF produced by tumor-MSCs is supported by an* in vivo* study from Suzuki et al. work, showing that an increased rate in metastasis in Lewis lung cancer model is related to an MSC induced neovascularization in these tumors [44].

Taken together, there is strong evidence that MSCs are able to migrate to solid tumors by the help of chemoattractants. It remains a largely unsolved question to what extent BM-MSCs quantitatively contribute to the stroma and what other tissues are involved in providing cellular support for the tumor mesenchyme.

A relatively recent concept proposes distinguishing between two MSC types (MSC1 and MSC2) which, in close analogy to the macrophage type 1 or type 2, represent a physiological, nontumor propagating phenotype (MSC1) and a tumor-promoting phenotype (MSC2). Experimentally, the induction of both subtypes could be achieved by the stimulation of Toll-like receptors 3 and 4 (TLR3, TLR4). The classification into the two phenotypes is mainly based on a distinct cytokine profile which includes an overexpression of TGF-*β* and its downstream effectors SMAD3 and SMAD4 [45, 46]. Although first published only in the* in vitro* context* in vivo* experiments confirm the different functions of MSC1 and MSC2, further evidence is needed to confirm the pathological role of this polarization and to validate the existence of both subtypes in tumors [45, 47].

**Figure 1:**
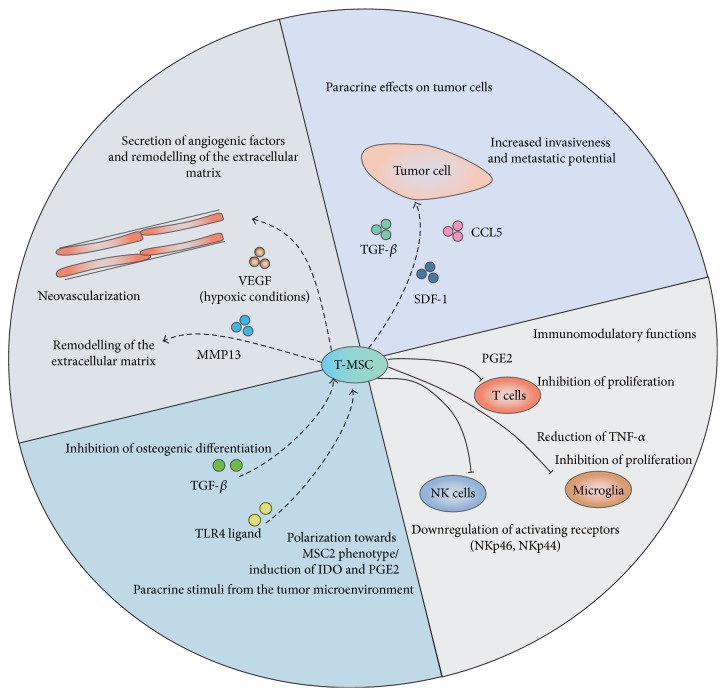
Interplay between T-MSC and the tumor microenvironment.

In summary, there are manifold mechanisms by which MSCs seem to exert their protumorigenic effect. They include (a) an inhibition of immune cells that are attracted to tumors as sites of chronic inflammation, (b) an induction of neovascularization that can promote tumor spread, (c) a transdifferentiation to myofibroblasts that contribute to stroma niche, (d) and lastly the remodeling of the extracellular matrix with the help of, for example, matrix metalloproteinases (as highlighted in Figure 1).

## 8. Evidence for Tumor-Inhibiting Properties of MSCs

Although the onus of proof points towards MSCs as tumor propagating and not tumor-inhibiting cells, the effect of MSCs may be context-depending. In fact, there are settings in which MSCs may abrogate tumor growth: When being injected into rat gliomas, MSCs are able to increase the therapeutic benefit of an immunotherapy with IFN-*γ*. This could be correlated to a stronger, antitumoral CD8^+^ T cell response against tumor cells [48]. Likewise, the same therapy enhancing effects of MSCs, when being coadministered with cisplatin, could be shown in a melanoma model [49].

In Kaposi's sarcoma, cell-cell contact was necessary for MSCs to slow down tumor growth. Akt kinase was implicated in this process as its phosphorylation in cancer cells decreases upon coculture with MSCs [32]. Similar observations could be made in pancreatic cancer where the administration of MSCs retarded tumor growth [50].

In pancreatic cancer, the inhibition of tumor growth was potentiated when transducing MSCs with IFN-*β*, confirming their suitability as a vector for immunotherapy [50]. Although these findings of a tumor inhibition by MSCs are conflicting in the case of, for example, glioblastoma, part of the divergence may be explained by different experimental settings: The choice of the* in vitro* and* in vivo* models may have a strong impact on the interaction between MSCs and the respective cancer. Thus, conducting more comparative studies using the same models and similar experimental conditions may help to reconcile these contradictions.

## 9. Multipotent Mesenchymal Stromal Cells as Possible Cells of Origin for Mesenchymal Tumors?

MSCs that are isolated from neoplastic tissues are typically considered to be devoid of the genetic aberrations that characterize the respective cancer [51–53].

Nonetheless there is increasing evidence that MSCs are able to serve as progenitor cells for certain, mainly soft tissue, tumors: The experimental silencing of the Ewing sarcoma specific EWS-FLI1 fusion transcript could partially restore the adipogenic differentiation potential of an ES tumor cell line, a property which the tumor cells do not display under native conditions. On a transcriptomic level, the expression profiles of these EWS-FLI1 silenced cells show a high degree of similarity to the gene expression profiles of MSCs [54].

Along a similar line, Tanaka et al. could detect an abundance of mesenchymal progenitors in the embryonic superficial zone of mouse that could give rise to Ewing sarcomas when transduced with the EWS-FLI1 fusion transcript [55].

Aside from Ewing sarcomas, the tumorigenesis of other sarcomas has also been linked to the presence of MSCs: MSCs from a p53 −/− background can serve as cell of origin for leiomyosarcoma and osteosarcoma [56].

Taken these evidences together, although MSCs within solid tumors may preserve their cytogenetic integrity, the similarity between the aforementioned tumors and MSCs at least indirectly points to MSCs as a potential paternal cell of origin of mesenchymal tumors such as Ewing sarcoma.

Table 5 synoptically catalogues entities in which tumor propagating or tumor-inhibiting properties of MSCs could be stated or for which MSCs have been implied as a putative cell of origin.

## 10. Conclusions

Although MSCs from the bone marrow and other “classical” sources have been characterized extensively, the phenotypic and functional properties of MSCs from tumors are poorly understood. This is in part due to the hitherto unclear distinction of these cells from tumor associated fibroblasts which share phenotypic markers and may also exert similar functions.

While the identity of tumor derived MSCs remains controversial and the number of publications that refer to tumor derived MSC directly remains small, a plethora of experiments studies the interaction of BM or T-MSCs and tumor cells both,* in vitro* and* in vivo*. The immunosuppressive function of both types of MSCs has been validated extensively and the cytokines which are implicated into mediating the effect seem to be identical between different MSC types.

The number of publications reporting a protumorigenic role of BM-MSCs/T-MSCs outweighs the ones which show antitumorigenic effects. Furthermore, the latter often refer to genetically engineered MSCs or combination of MSCs with an additional therapeutic agent and as such do not consider the native situation [57].

It is thus conceivable that the divergent findings on the role of MSCs in tumors may partly be due to the different settings that were used for the experiments. Moreover, the source of MSCs may influence their effect on tumor growth: Akimoto et al. demonstrated that adipose tissue derived MSCs could promote glioblastoma growth* in vitro* and* in vivo* while umbilical cord-blood derived cells inhibited the tumor progression [58].

It is noteworthy that in a given tumor entity only very few conflicting reports on tumor-inhibiting and tumor propagating effects of MSCs have been shown. This may hint at MSC-effects that are depending not only on the origin of the MSCs but also on the entity and context that is studied. To resolve the role of MSCs in tumorigenesis, more comparative examinations using identical settings between different entities are needed.

On the basis of these investigations, a final judgment on the role of MSCs may possibly be achieved; this would be highly desirable given the increasing number of clinical trials banking on the therapeutic use of BM-MSCs.

## Figures and Tables

**Table 1:** Overview on cell types that are present within the tumor microenvironment (based on [7, 59]).

Cell type	Function and contribution to the tumor microenvironment	Factors which contribute to the function
Neutrophil granulocytes	(i) Remodelling of extracellular matrix (ii) Promotion of tumor growth, angiogenesis, and metastasis	(i) MMP (ii) VEGF, TGF-*β*

T cells	(i) Functionally compromised in the tumor microenvironment (ii) Immunosuppression	IL-10, TGF-*β*

NK cells	Tumor cell lysis (often immature NK cells infiltrating the tumor)	(Reduced) NKp44/NKp33 expression in tumors

Dendritic cells	Skewed towards immunosuppression (induce regulatory T cells)	TGF-*β*, IL-10

Tumor associated macrophages	(i) Functionally compromised in the tumor microenvironment (ii) Mostly polarized towards the M2 phenotype (iii) Inhibition of immune cell proliferation	IL-4 (induces M2 phenotype)

Mesenchymal cells/tumor associated fibroblasts	(i) Secretion of tumor growth promoting factors (ii) Remodelling of extracellular matrix	(i) TGF-*β*, HGF, VEGF (ii) MMP

Endothelial cells/blood vessels	(i) Secretion of VEGF (ii) Formation of new blood vessels	VEGF, PDGF-*α*

Tumor cells	(i) Immunosuppression (ii) ECM remodeling (iii) Cross-talk with TAF/MSCs and induction	MMP, IL-10, IL-6

**Table 2:** (Positive) Phenotypic markers and features for both MSCs and TAFs based on [15, 60–63]. Markers for which presence in both TAFs and MSCs has been demonstrated are shown in the middle while markers which have only been demonstrated in either one of the cell types are presented in the left or right column.

MSCs	Tumor associated fibroblasts
**Minimal criteria for the definition of MSCs**	
CD73, CD90, CD105	
(according to ISCT)	

**Extracellular matrix proteins**	
tenascin-c, thrombospondin-1, periostin	

**Adhesion molecules/lineage markers**	
HCAM, VCAM-1, MCAM, LCAM, integrin-*β*1,	

**Growth**	
**factors/hormones**	
SDF-1, NPPB, FGF, VEGF, FGFR3	

**Immunological markers**	
HLA-ABC	

**Various other markers**	**Activation marker**
CD44, CD271, CD71, CD106, CD146, MSCA-1	FAP

**Table 3:** Immunomodulation by MSCs and TAFs.

Tumor entity	Effects observed	Effects mediated by	Literature
Cervical cancer	Downregulation of HLA-I in cervical cancer by T-MSCs cell lines and reduced cytolysis by CTL-cells	IL-10	[26]

Melanoma	Impairment of NK cell answer against melanoma cells by reduced upregulation of NKp44, NKp33, and DNAMI after exposure to T-MSCs	(i) Reduction of NKp44 and expression (mediated by PGE2) (ii) Reduction of DNAMI expression (depending on cell to cell contact)	[64, 65]

Pancreatic cancer	Depletion of arginine renders tumor infiltrating T cells dysfunctional	Expression of ARG2 and arginine depletion	[66]

NSCLC	CD3/CD28 depending activation of T cells	IL-6	[67]

Follicular lymphoma	MSCs from follicular lymphoma patients display increased recruitment of TAM and a distinct gene expression profile	Overexpression of CCL2	[68]

**Table 4:** Overview on homing mechanisms of MSCs and MSC effect on tumor growth.

Entity	Experimental design	Effects observed	Literature
NSCLC	*In vitro* coculture study of MSCs and tumor cells	Interaction of MIF with CXCR4/SDF-1 contributes to MSCs homing	[69]

Melanoma	*In vivo* homing of cytosine deaminase expressing MSCs in subcutaneous melanoma	MSCs abrogate tumor growth by TNF-*α* production	[69, 70]

Breast cancer	(i) *In vitro *and *in vivo* migration of breast cancer cells to the bone marrow being facilitated by BM-MSC (ii) Coinjection of BM-MSCs and breast cancer cells	Tac1 mediated entry of breast cancer cells to the bone marrow	[38]

Neuroblastoma	*In vitro* migration of MSCs towards neuroblastoma cell lines	Migration of MSCs depending on uPA expression	[71]

Glioblastoma/brainstem glioma	(i) *In vivo* homing of MSCs in GL216 glioma model	(i) Presence of MSCs in the GL216 glioma model validated the homing process, change of the phenotype of tumor cells due to MSC influence; CXCR4 and CXCR6 contribute to the homing of MSCs	[72]
	(ii) *In vitro* migration of MSCs towards GBM cell lines	(ii) *In vitro* migration was mediated by HGF	[73]

Mesothelioma	*In vivo* homing of TNF-*α* overexpressing MSCs in intraperitoneal mesothelioma	*In vitro* and *in vivo* induction of apoptosis in mesothelioma cells	[74]

Hepatocellular carcinoma	(i) *In vivo* homing of MSCs in HCC	(i) MSCs primed with AMF displayed increased migratory capability to HCC and reduced MMP2 expression	[75]
	(ii) Coculture of MSCs and HCC cell lines	(ii) Increased invasiveness of HCC due to CCL5	[76]

Multiple myeloma	Decreased survival of mice after MSCs and multiple myeloma cell infusion	CCL25 production by MM cells as a chemoattractant	[77]

Gastric cancer	Recruitment of BM-MSCs to gastric cancer	CXCR4/SDF-1 axis	[30]

Pancreatic cancer	Transplantation of bone marrow (BM) cells into sublethally irradiated SCID mice and subcutaneous transplantation of a pancreatic cancer cell line; assessment of stromal cell contribution by BM-MSCs	High frequency of BM-derived myofibroblasts in the tumor stroma	[78]

**Table 5:** Studies in which MSCs have been implicated as cell of origin for specific tumors or have been shown to display protumorigenic or antitumorigenic effects.

MSCs as possible cell of origin	Tumor propagating effects of MSCs	Tumor inhibiting effects of MSCs
Ewing sarcoma [79]	Breast cancer [35]	Hepatoma [79]
Osteosarcoma [80]	Ovarian cancer [80]	Kaposi's sarcoma [81]
Leiomyosarcoma [80]	Head and neck squamous cancer [82]	Glioblastoma [58]
Synovial sarcoma [83]	Colon cancer [84]	Pancreatic carcinoma [50]
	Osteosarcoma [85]	Glioblastoma [86]
	Melanoma [87]	
	Hepatocellular carcinoma [88]	
	Glioblastoma [58]	

## Abbreviations

*α*-SMA: *α*-smooth muscle actin
AMF: Autocrine motility factor
BM-MSC: Bone marrow derived multipotent mesenchymal stromal cell
CCL5: Chemokine ligand 5
DNAMI: DNAX accessory molecule-1
FAP: Fibroblast activation protein
FLI1: Friend leukemia integration 1
HCC: Hepatocellular carcinoma
HGF: Hepatocyte growth factor
IDO: Indoleamine 2,3-dioxgynease
IFN-*β*: Interferon *β*
IL: Interleukin
ISCT: International Society for Cellular Therapy
MIF: Macrophage inhibitory factor
MM: Multiple myeloma
MSC: Multipotent mesenchymal stromal cell
MSCA-1: Mesenchymal stem cell antigen-1
NPPB: Brain natriuretic peptide
NSCLC: Nonsmall cell lung cancer
SDF-1: Stromal derived factor 1
SMAD3: Mothers against DPP homologue 3
SMAD4: Mothers against DPP homologue 4
SSEA: Stage specific embryonic antigen A
TAC1: Protachykinin 1
TGF-*β*: Transforming growth factor *β*
TLR: Toll-like receptor
TRAIL: TNF-related apoptosis inducing ligand
TNF-*α*: Tumor necrosis factor *α*
uPA: Urokinase plasminogen activator
VCAM: Vascular cell adhesion protein-1
VEGF: Vasoendothelial growth factor.
